# Genetic Deletion of Uncoupling Protein 3 Exaggerates Apoptotic Cell Death in the Ischemic Heart Leading to Heart Failure

**DOI:** 10.1161/JAHA.113.000086

**Published:** 2013-06-21

**Authors:** Cinzia Perrino, Gabriele G. Schiattarella, Anna Sannino, Gianluigi Pironti, Maria Piera Petretta, Alessandro Cannavo, Giuseppe Gargiulo, Federica Ilardi, Fabio Magliulo, Anna Franzone, Giuseppe Carotenuto, Federica Serino, Giovanna G. Altobelli, Vincenzo Cimini, Alberto Cuocolo, Assunta Lombardi, Fernando Goglia, Ciro Indolfi, Bruno Trimarco, Giovanni Esposito

**Affiliations:** 1 Department of Advanced Biomedical Sciences, Federico II University, Naples, Italy (C.P., G.G.S., A.S., G.P., M.P.P., A.C., G.G., F.I., F.M., A.F., G.C., F.S., G.G.A., V.C., A.C., B.T., G.E.); 2 Department of Biology, Federico II University, Naples, Italy (A.L.); 3 Department of Biology Sciences, Geology and Environment, Sannio University, Benevento, Italy (F.G.); 4 Department of Cardiology, Magna Graecia University, Catanzaro, Italy (C.I.)

**Keywords:** cardiac remodeling, free radicals, mitochondria, uncoupling protein

## Abstract

**Background:**

Uncoupling protein 3 (ucp3) is a member of the mitochondrial anion carrier superfamily of proteins uncoupling mitochondrial respiration. In this study, we investigated the effects of ucp3 genetic deletion on mitochondrial function and cell survival under low oxygen conditions in vitro and in vivo.

**Methods and Results:**

To test the effects of *ucp3* deletion in vitro, murine embryonic fibroblasts and adult cardiomyocytes were isolated from wild‐type (WT, n=67) and ucp3 knockout mice (ucp3^−/−^, n=70). To test the effects of ucp3 genetic deletion in vivo, myocardial infarction (MI) was induced by permanent coronary artery ligation in WT and ucp3^−/−^ mice. Compared with WT, ucp3^−/−^ murine embryonic fibroblasts and cardiomyocytes exhibited mitochondrial dysfunction and increased mitochondrial reactive oxygen species generation and apoptotic cell death under hypoxic conditions in vitro (terminal deoxynucleotidyl transferase‐dUTP nick end labeling–positive nuclei: WT hypoxia, 70.3±1.2%; ucp3^−/−^ hypoxia, 85.3±0.9%; *P*<0.05). After MI, despite similar areas at risk in the 2 groups, ucp3^−/−^ hearts demonstrated a significantly larger infarct size compared with WT (infarct area/area at risk: WT, 48.2±3.7%; ucp3^−/−^, 65.0±2.9%; *P*<0.05). Eight weeks after MI, cardiac function was significantly decreased in ucp3^−/−^ mice compared with WT (fractional shortening: WT MI, 42.7±3.1%; ucp3^−/−^ MI, 24.4±2.9; *P*<0.05), and this was associated with heightened apoptotic cell death (terminal deoxynucleotidyl transferase‐dUTP nick end labeling–positive nuclei: WT MI, 0.7±0.04%; ucp3^−/−^ MI, 1.1±0.09%, *P*<0.05).

**Conclusions:**

Our data indicate that ucp3 levels regulate reactive oxygen species levels and cell survival during hypoxia, modulating infarct size in the ischemic heart.

## Introduction

Heart failure (HF) represents one of the main causes of death and mortality in the Western countries.^1^ Coronary artery disease and arterial hypertension represent the leading causes of HF.^2^

Oxidative stress mediated by reactive oxygen species (ROS) plays a striking role in cardiomyocyte (CM) death and in the pathogenesis of HF, particularly after myocardial infarction (MI).^3–4^ Although antioxidants attenuate cardiac remodeling in different experimental models of MI,^5^ and several drugs that have beneficial effects on ventricular function and prognosis also have some antioxidant effects,^6–7^ clinical trials have not confirmed the beneficial effects of different antioxidants on postischemic cardiac remodeling.^8–9^

Increased ROS production may contribute to the development of cardiac dysfunction with different mechanisms, including myocyte loss via apoptosis or other cell death mechanisms.^10–12^ Several sources of increased ROS production have been identified in the overloaded heart,^13^ with the mitochondria being one of the major components.^13^ Mitochondrial ROS production can be controlled by a family of uncoupling proteins (ucps) that catalyze a regulated proton leak across the inner mitochondrial membrane, diverting free energy from the ATP synthesis chain to the production of heat,^14^ and thus reducing ROS production.^15^

So far, 5 ucp isoforms have been identified in mammals, named ucp1 to 5 in the order of their discovery^16^; ucp1 is the most extensively characterized member of the family and is exclusively expressed in brown adipose tissue.^17–18^ In contrast, ucp2 and ucp3 are expressed primarily in skeletal muscle and in the heart, where their role is still not completely defined. Coronary artery ligation and in vitro hypoxia are potent inducers of both ucp2 and ucp3 messenger RNA (mRNA) and protein levels, suggesting that these proteins might play a relevant role in the regulation of cardiac tolerance to ischemia. Indeed, recent studies have shown that ucp2 or ucp3 activation might mitigate ROS production^14,16,19–20^ and CM death.^21^ In particular, it has been proposed that enhanced ucp2 expression might represent an important adaptation in the chronically ischemic myocardial tissue.^22^ In contrast, ucp3 expression is significantly reduced in samples from failing human hearts, and mechanical unloading of the left ventricle has been shown to reverse these changes.^23^

In the present study, we tested whether ucp3 deletion might affect mitochondrial function, ROS production, and cell survival in vitro under normal or hypoxic conditions and in the heart undergoing chronic HF after permanent coronary artery ligation. Our study shows that ucp3 genetic deletion promotes mitochondrial dysfunction and increases ROS production and apoptotic cell death during in vitro hypoxia and in the ischemic heart, suggesting that ucp3 might represent a novel important determinant of infarct size, postischemic cardiac remodeling, and survival.

## Materials and Methods

### Animal Studies

All experiments involving animals conformed to the “Guide for the Care and Use of Laboratory Animals” published by the US National Institutes of Health (NIH Publication No. 85‐23, revised 1996) and were approved by the animal welfare regulation of University Federico II of Naples, Italy. Mice were purchased from the Jackson Laboratory (genetic background—Strain: 129S4/SvJae). The ucp3^−/−^ mice were obtained as previously described.^24^ Male adult ucp3^−/−^ (aged 8 to 9 weeks, N=70) and wild‐type (WT) mice (aged 8 to 9 weeks, N=67) were included in the study and maintained under identical conditions of temperature (21±1°C), humidity (60±5%), and light–dark cycle and had free access to normal mouse chow.

### Mouse Model of MI and Infarct Size Determination

MI was induced in ucp3^−/−^ (n=30) and WT mice (n=28) by permanent coronary artery ligation as previously described.^25^ Sham‐operated animals underwent the same procedure without ligation of the left coronary artery at the same time (sham: WT, n=11; ucp3^−/−^, n=12). An additional group of mice underwent systemic delivery of the antioxidant α‐tocopherol 12 hours before, 75 minutes before, and 12 hours after MI (MI+α‐tocopherol, 150 mg/kg IV, n=5). To determine infarct size, some animals were anesthetized 24 hours (WT, n=7; ucp3^−/−^, n=7) after coronary artery ligation, and the hearts were perfused with 1% Evans blue to determine the area at risk (AAR) and then removed, as previously described.^26^ Each heart was sliced horizontally to yield 4 slices, and the slices were incubated in 1% triphenyltetrazolium chloride (Sigma‐Aldrich) prepared with Tris 200 mmol/L buffer (pH 7.8) for 15 minutes at 37°C. With this procedure, viable nonischemic myocardium stains blue, ischemic but still viable myocardium stains red, whereas the necrotic myocardium does not stain and appears pale white. The infarct area (IA, white) and the AAR (red and white) from each section were measured using an image analyzer (ImageJ software). Ratios of AAR/left ventricular (LV) area and IA/AAR were calculated and expressed as a percentage. Eight weeks after coronary artery ligation, in additional mice the hearts were arrested in diastole with a 300‐μL injection of saturated KCl solution in the right atrium, and the hearts were flash‐frozen in liquid nitrogen to perform molecular analyses or fixed in 4% formaldehyde to perform histological analyses (see later).

### Mouse Model of Myocardial Ischemia–Reperfusion Injury

Myocardial ischemia–reperfusion injury was induced in ucp3^−/−^ (n=6) and WT mice (n=6) as previously described.^27^ Briefly, ucp3^−/−^ mice and their WT littermates were anesthetized with an intramuscular injection of ketamine 100 mg/kg and xylazine 2.5 mg/kg. After intubation (polyethylene‐60 tubing), the animals were ventilated with a stroke rate of 130/min and a tidal volume of 1 mL. A midline thoracotomy and pericardiotomy were performed. The left anterior descending coronary artery was occluded 2 to 3 mm distal to the tip of the left auricle using a 7.0 silk suture and a small tube to form a snare for 30 minutes. Hearts were then reperfused for 120 minutes. At the end of the protocol, the hearts were freshly put on mitochondria isolation buffer (in mmol/L: sucrose 70, mannitol 220, Tris 20 (pH 8.5, EDTA 1, pH 7.4) to perform mitochondrial respiration experiments (see later).

### Morphological Studies

Mouse heart specimens fixed in 4% formaldehyde were embedded in paraffin, and after deparaffinization and rehydration, 4‐μm‐thick sections were prepared, mounted on glass slides, and stained with hematoxylin–eosin or Masson's trichrome as previously described.^28^ To determine infarct size, total LV epicardial and endocardial circumferences and epicardial and endocardial borders of infarcted regions were measured and averaged using computer‐assisted image analysis software (ImageJ software). To quantify myocardial capillary density, lectin staining was used to specifically stain endothelial cells, as previously described.^29^

The DNA nicks were determined with the use of an in situ Apoptosis Detection kit or ApopTag Fluorescein Direct in Situ Apoptosis Detection kit (Chemicon) according to the manufacturer's instructions as previously described both in vivo and in cultured cells (see later).^30–31^ Terminal deoxynucleotidyl transferase‐dUTP nick end labeling (TUNEL) staining was visualized by specific green fluorescence and nuclei by 4′‐6‐diamidino‐2‐phenylindole (DAPI). The number of TUNEL‐positive CM nuclei was counted, and data were normalized per total nuclei identified by DAPI staining in the same sections (n=7 or 8 animals/group). Equal numbers of cells were analyzed per group, and statistical analysis was performed on myocyte groups by 2‐way ANOVA.

### Transthoracic Echocardiography

Cardiac function was noninvasively monitored by transthoracic echocardiography using the Vevo 770 high‐resolution imaging system (VisualSonics) before the surgery and right before termination, 8 weeks after surgery, as previously described.^32^ Briefly, the mice were anesthetized with an intramuscular injection of ketamine 100 mg/kg and xylazine 2.5 mg/kg, and echocardiograms were performed with a 30‐MHz RMV‐707B scanning head.

### Positron Emission Tomography

The accuracy and reproducibility of high‐resolution positron emission tomography/computed tomography with 2‐deoxy‐2[^18^F]fluoro‐d‐glucose (^18^F‐FDG) for noninvasive quantification of myocardial infarct size in mice have been recently documented.^33^ Eight weeks after surgical coronary ligation, ^18^F‐FDG in 100 mL of 0.9% saline was injected intravenously, to obtain images of mouse hearts, 1 hour before each scan. After the administration of ketamine 100 mg/kg and xylazine 2.5 mg/kg, the mouse was placed on a heating pad to maintain a body temperature within the normal range. List‐mode data were acquired for 15 minutes and subsequently reconstructed into a single image volume of 110×60×60 mm^3^. The images were obtained using an eXplore Vista computed tomograph (General Electric). The animals had unrestricted access to water and their normal food before scanning.

### Cell Culture and In Vitro Hypoxia

Mouse embryonic fibroblasts (MEFs) and adult mouse CMs were obtained from WT and ucp3^−/−^ mice.

For MEF isolation, the embryos were taken from pregnant females at days 13 and 14 of gestation. Immediately after the levy, embryos were transferred in new sterile plates containing PBS–penicillin/streptomycin 2%. After removal of the head and the liver, embryos were repeatedly washed in a sterile solution made of PBS–penicillin/streptomycin 2% and then transferred in a 1‐mL syringe containing trypsin‐EDTA. Hence, embryos were mechanically homogenized and the product was incubated at 37°C for 5 minutes. The isolated cells were maintained in Dulbecco's modified Eagle's medium (DMEM) supplemented with 0.1% FBS, l‐glutamine 200 mg/mL, penicillin 100 units/mL, streptomycin 100 μg/mL, and 0.01% nonessential amino acids.

CMs were isolated and cultured using a modification of the collagenase dissociation method described by Zhou et al.^34^ Briefly, mice were treated with heparin (50 units) and anesthetized with an intramuscular injection of ketamine 100 mg/kg and xylazine 2.5 mg/kg. The heart was quickly excised, and the aorta was cannulated for retrograde perfusion in a Langendorff apparatus at a constant flow rate of 3 mL/min at 37°C. The heart was perfused for 9 to 10 minutes with isolation buffer (NaCl 120 mmol/L, KCl 5.4 mmol/L, MgSO_4_ 1.2 mmol/L, NaH_2_PO_4_ 1.2 mmol/L, glucose 5.6 mmol/L, NaHCO_3_ 5 mmol/L, HEPES 10 mmol/L, CaCl_2_ 50 μmol/L, 2,3‐butanedione monoxime [BDM] 10 mmol/L, and taurine 5 mmol/L), followed by digestion for 9 minutes with collagenase II (1.5 mg/mL; Worthington) in isolation buffer. After digestion, the soft and flaccid heart was removed, and the myocytes were suspended in isolation buffer. A series of 4 centrifugations (40*g*×1 minute) and resuspensions were used for stepwise Ca^2+^ reintroduction from 50 μmol/L to 1.0 mmol/L, which was the final medium Ca^2+^ concentration. Isolated CMs were plated for 2 hours onto 35‐ and 60‐mm tissue culture dishes coated with laminin 10 μg/mL. The cells were suspended in minimum essential medium (MEM) with Hanks' buffered salt solution (HBSS), penicillin 10 μg/mL, vitamin B12 1.5 μmol/L, and BDM 10 mmol/L. After this period of attachment, the medium was changed to MEM‐HBSS containing penicillin 10 μg/mL, vitamin B12 1.5 μmol/L, and BDM 1 mmol/L and was incubated overnight at 37°C in a humidified atmosphere of 1% CO_2_ and air. The culture protocol yielded an average of 80% rod‐shaped myocytes at a plating density of 50 cells/mm^2^ that were viable at pH 7.2 for 48 hours. Experiments were performed the day after isolation and culture.

Hypoxia (2% O_2_) was induced when MEFs and CMs were at 0.85 confluence. After being washed 3 times, cultures were transferred to a 37°C incubator within an hypoxic chamber (93% nitrogen–5% CO_2_–2% oxygen), and DMEM was replaced with a saline buffer that was prebubbled for 5 minutes with the same gas mix to provide a media Po_2_ (14.7 mm Hg) equivalent to that in the ambient air of the chamber.

### Mitochondrial Dehydrogenase Assay

Cell viability of both WT and ucp3^−/−^ MEFs was assessed by measuring the activity of the mitochondrial dehydrogenase under basal conditions and after 24‐hour hypoxia. Cells were plated onto 12 wells, and then the conversion of 3‐(4,5‐dimethylthiazol‐2‐yl)‐2,5‐diphenyltetrazolium bromide (MTT) to formazan was measured by optical density at 490 nm with a spectrophotometer (SmartSpec Plus; Bio‐Rad).

### Mitochondrial Aconitase Activity Assay

Changes in mitochondrial aconitase activity were detected using a BIOXYTECH Aconitase‐340 kit (OxisResearch) according to the manufacturer's instructions. Mitochondrial protein fraction was obtained as previously described.^31^ Briefly, LV samples were lysed in a buffer containing mannitol 250 mmol/L, EGTA 0.5 mmol/L, HEPES 5 mmol/L, and 0.1% BSA. Samples were then centrifuged at 1000*g* for 5 minutes at 4°C to pellet nuclei, membranes, and unbroken cells. The supernatant was centrifuged at 10 500*g* for 15 minutes at 4°C to separate the soluble cytosolic fraction from a pellet containing mitochondria. The pellet was sonicated and then resuspended in lysis buffer. The enrichment for mitochondria in the membrane fraction and their absence in the soluble cytosolic fraction were assessed by immunoblotting for the mitochondrial voltage‐dependent anion channel. Aconitase activity was expressed as microunits of *cis*‐aconitate converted per minute per milligram of mitochondrial protein.

### Determination of Mitochondrial Respiration

Mitochondrial respiration was measured polarographically by using a Clark‐type oxygen electrode (Rank Brothers) at 25°C in 0.5 mL of respiratory buffer (KCl 80 mmol/L, HEPES 50 mmol/L, EGTA 1 mmol/L, KH_2_PO_4_ 5 mmol/L, MgCl_2_ 2 mmol/L, 0.5% BSA, pH 7.0). Respiration was initiated by adding glutamate 5 mmol/L and malate 2.5 mmol/L as substrates for complex I or succinate 5 mmol/L (in combination with rotenone 2 μmol/L to inhibit complex I) as substrate for complex II. After recording of basal oxygen consumption, respiration was determined after the addition of ADP 200 μmol/L (state 3 ADP). State 3 ADP was terminated by adding the ATP synthase inhibitor oligomycin (1 μg/mL) to achieve a state 4_O_ rate (to prevent ATP recycling contribution to the respiration). Complex IV respiration was determined after the addition of antimycin A 1.8 μmol/L to inhibit complex II and TMPD (*N,N,N0*,*N*0‐tetramethyl‐*p*‐phenylenediamine) 300 μmol/L and ascorbate 3 mmol/L, which donates electrons to cytochrome oxidase via the reduction of cytochrome c.

### NAD^+^/NADH Assay

The total levels of nicotinamide adenine dinucleotide (NADt) and its reduced form, NADH, were analyzed in cell and heart lysates using a NAD^+^/NADH quantification kit (MBL; International Corporation) according to the manufacturer's instructions. NADt or NADH levels were expressed in picomoles per 10^6^ cells (in MEFs) or nanograms per milligrams of protein (in cardiac lysates). NAD/NADH ratio is calculated as NADt−NADH/NADH.

### Mitochondrial Membrane Potential Assessment by Tetramethylrhodamine Ethyl Ester

Mitochondrial membrane potential (Δψ_**m**_) was assessed by flow cytometry using tetramethylrhodamine ethyl ester (TMRE; Molecular Probes), a nontoxic monovalent cation that reversibly accumulates in the mitochondrial lipid environment according to membrane potential with a Nernstian distribution. After specific treatments, cultured WT and ucp3^−/−^ MEFs or CMs were resuspended in their complete media and incubated with TMRE 50 nmol/L for 20 minutes in the dark at 37°C. At the end of incubation, cells were resuspended in the flow analysis buffer (PBS 1×), and kept on ice until analysis.

### Mitochondrial ROS Generation

Mitosox Red (Molecular Probes) was used to assess the generation of mitochondrial superoxide in vivo and in vitro as previously described.^31^ Ten micrograms of Mitosox Red in 200 μL of PBS was injected into the tail vein of each mouse. Animals were killed 90 minutes later, the hearts were rapidly removed, and the left ventricle was dissected and fixed overnight in 4% paraformaldehyde, included in optimum cutting temperature compound (Miles Pharmaceuticals), and snap frozen in liquid nitrogen. Then, 10‐μm LV sections were cut using a cryostat. In vitro, cultured WT and ucp3^−/−^ MEFs or CMs under normoxic and hypoxic conditions were incubated with the Mitosox Red reagent for 10 minutes and then visualized with use of a fluorescent Nikon Eclipse TE 2000‐U microscope.

### Electron Microscopy

In electron microscopy studies, 6 animals (sham: ucp3^−/−^, n=3; WT, n=3; MI: ucp3^−/−^, n=3; WT, n=3) were anaesthetized with an intramuscular injection of ketamine 100 mg/kg and xylazine 5 mg/kg, and the hearts were fixed by retrograde aortic perfusion. The hearts were then dissected, and the LV anterior wall was selected for the detailed comparative study reported here. Tissue samples were cut into small blocks and postfixed with the same fixative for 2 hours and then with 2% osmium tetroxide for 1 hour. They were embedded in araldite at 60°C. Ultrathin sections mounted on grids were counterstained with uranyl acetate and lead citrate.

### RNA Extraction and Real‐Time PCR

Total RNA was obtained from ventricular specimens and from MEFs as previously described.^31^ Oligo‐dT first‐strand cDNA synthesis and ucp2 and ucp3 mRNA expression were determined in cardiac samples and in normoxic/hypoxic MEFs by real‐time quantitative PCR (RT‐PCR) using an IQ‐5 Multicolor Real‐Time PCR Detection System (Bio‐Rad).

### Protein Extraction and Immunoblot Analysis

Cultured cells and LV samples were lysed, and immunoblotting was performed using commercially available antibodies. Cultured cells and LV samples were lysed in a buffer containing NaCl 150 mmol/L, Tris 50 mmol/L (pH 8.5), EDTA 1 mmol/L, 1% v/v Nonidet P‐40, 0.5% w/v deoxycholate, NaF 10 mmol/L, Na_2_ pyrophosphate 10 mmol/L, PMSF 2 mmol/L, leupeptin 2 μg/mL and aprotinin 2 μg/mL. Lysates were incubated on ice for 15 minutes and then centrifuged at 38 000*g* for 30 minutes at 4°C. Protein concentrations in all lysates were measured using a dye‐binding protein assay kit (Bio‐Rad) and a spectrophotometer reader (Bio‐Rad) at a wavelength of 595 nm. Immunoblotting was performed using the following commercially available antibodies: cleaved caspase 3 (rabbit monoclonal; Cell Signaling), phosphorylated‐AKT (rabbit polyclonal; Santa Cruz), p53 (mouse monoclonal; Santa Cruz), UCP2 (goat polyclonal; Sigma‐Aldrich), UCP3 (rabbit polyclonal; Sigma‐Aldrich), total AKT (rabbit polyclonal; Santa Cruz), GAPDH (mouse monoclonal; Upstate Biotechnology), and α‐tubulin (mouse monoclonal; Santa Cruz). Secondary antibodies were purchased from Amersham Life Sciences. Bands were visualized by use of enhanced chemiluminescence (ECL; Amersham Life Sciences) according to the manufacturer's instructions and were quantified using densitometry (Chemidoc; Bio‐Rad). Each experiment and densitometric quantification was separately repeated at least 3 times.

### Statistical Analysis

Data are expressed as mean±SE. Comparisons between 2 groups were performed using the unpaired Student *t* test. For MI experiments, comparisons were made by 2‐way analysis of variance (ANOVA) or, when noted, by 1‐way ANOVA, and *P* values shown indicate the effect of genotype on the MI‐stimulated response. Correction for multiple comparisons was made using the Student–Newman–Keuls method. A minimum value of *P*<0.05 was considered statistically significant. All the analyses were performed with GraphPad Prism version 5.01.

## Results

### Increased Oxidative Stress and Mitochondrial Dysfunction in Primary MEFs and Adult CMs From ucp3^−/−^ or WT Mice

**Figure 1.**
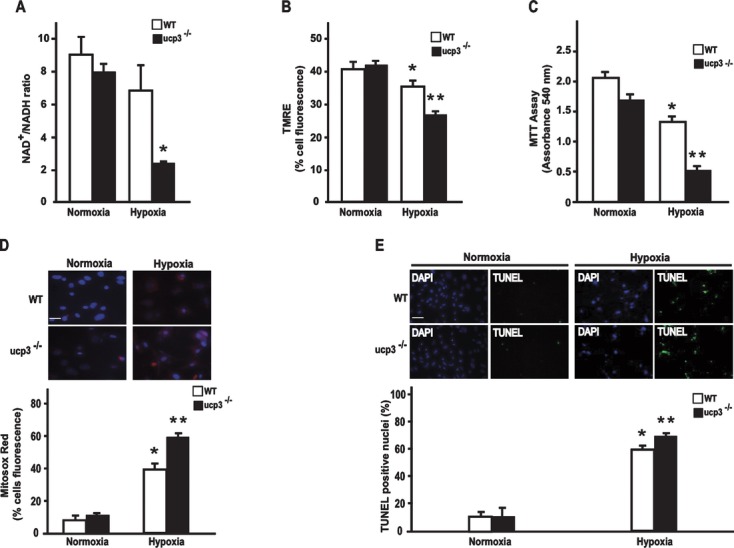
The ucp3 genetic deletion impairs mitochondrial activity, increases ROS production, and promotes cell death in MEFs. A, NAD^+^/NADH ratio in wild‐type (WT) and ucp3^−/−^ MEFs under normoxic or hypoxic conditions (*P<0.05 for 2‐way ANOVA test comparing normoxia and hypoxia of different genotype; *P<0.05 vs normoxia, WT hypoxia ). B, Mitochondrial membrane potential assessed by TMRE in WT and ucp3^−/−^ MEFs under normoxic or hypoxic conditions (*P<0.05 and **P<0.05 for 2‐way ANOVA test comparing normoxia and hypoxia of different genotypes; *P<0.05 vs normoxia; **P<0.05 vs normoxia, WT hypoxia). C, Bar graphs showing the conversion of yellow MTT to purple formazan in the mitochondria of WT and ucp3^−/−^ MEFs under normoxic or hypoxic conditions (*P<0.05 and **P<0.05 for 2‐way ANOVA test comparing normoxia and hypoxia of different genotype; *P<0.05 vs normoxia; **P<0.05 vs normoxia, WT hypoxia). D, Top: Representative images illustrating mitochondrial ROS production in MEFs from WT and ucp3^−/−^ mice under normoxic or hypoxic conditions. Scale bar=10 μm. Bottom: Cumulative data of multiple independent experiments (*P<0.05 and **P<0.05 for 2‐way ANOVA test comparing normoxia and hypoxia of different genotypes; *P<0.05 vs normoxia; **P<0.05 vs normoxia, WT hypoxia). E, Top: Representative DAPI and TUNEL staining in MEFs from WT and ucp3^−/−^ mice under normoxic or hypoxic conditions. Positive nuclei appear green (arrowheads). Bottom: Cumulative data of multiple independent experiments (*P<0.05 and **P<0.05 for 2‐way ANOVA test comparing normoxia and hypoxia of different genotype; *P<0.05 vs normoxia; **P<0.05 vs normoxia, WT hypoxia). Scale bar=250 μm. ucp3 indicates uncoupling protein 3; ROS, reactive oxygen species; MEFs, mouse embryonic fibroblasts; WT, wild‐type; ANOVA, analysis of variance; TMRE, tetramethylrhodamine ethyl ester; MTT, 3‐(4,5‐dimethylthiazol‐2‐yl)‐2,5‐diphenyltetrazolium bromide; DAPI, 4′‐6‐diamidino‐2‐phenylindole; TUNEL, terminal deoxynucleotidyl transferase‐dUTP nick end labeling.

**Figure 2.**
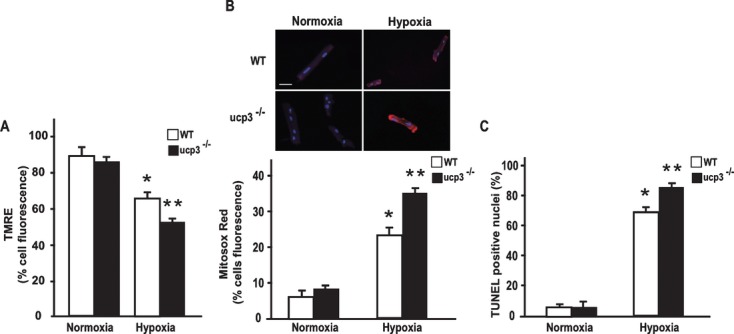
The ucp3 deletion impairs mitochondrial activity, increases reactive oxygen species (ROS) production, and promotes cell death in adult (CMs). A, Mitochondrial membrane potential assessed by TMRE in WT and ucp3^−/−^ CMs under normoxic or hypoxic conditions (*P<0.05 and **P<0.05 for 2‐way ANOVA test comparing normoxia and hypoxia of different genotype; *P<0.05 vs normoxia; **P<0.05 vs normoxia, WT hypoxia). B, Top representative images illustrating mitochondrial ROS production in CMs from WT and ucp3^−/−^ mice under normoxic or hypoxic conditions. Scale bar=10 μm. Bottom cumulative data of multiple independent experiments (*P<0.05 and **P<0.05 for 2‐way ANOVA test comparing normoxia and hypoxia of different genotype; *P<0.05 vs normoxia; **P<0.05 vs normoxia, WT hypoxia). Scale bar=250 μm. C. Cumulative data of multiple independent experiments to evaluate TUNEL staining in CMs from WT and ucp3^−/−^ mice under normoxic or hypoxic conditions (*P<0.05 and **P<0.05 for 2‐way ANOVA test comparing normoxia and hypoxia of different genotype; *P<0.05 vs normoxia; **P<0.05 vs normoxia, WT hypoxia). ucp3 indicates uncoupling protein 3; CM, cardiomyocyte; WT, wild‐type; ANOVA, analysis of variance; TMRE, tetramethylrhodamine ethyl ester.

Primary MEFs were isolated from WT and ucp3^−/−^ mice to analyze the effects of ucp3 genetic deletion on mitochondrial function, ROS production, and cell survival during normoxia or after 4 hours of hypoxia. Compared with WT, hypoxic ucp3^−/−^ MEFs exhibited more pronounced mitochondrial dysfunction, as shown by a significant reduction in the NAD^+^/NADH ratio (Figure 1A) and in mitochondrial membrane potential, measured as percent reduction of the TMRE fluorescence (Figure 1B). Mitochondrial dysfunction was also associated with a significant decrease in mitochondrial dehydrogenase (MTT) activity in ucp3^−/−^ MEFs compared with WT under both normoxic or hypoxic conditions (Figure 1C) and with a significant increase in mitochondrial production of ROS, as shown by Mitosox Red staining (Figure 1D). Mitochondrial dysfunction and increased ROS production may be responsible for the significantly increased apoptotic cell death induced by ischemia in ucp3^−/−^ MEFs (Figure 1E). Importantly, these results were confirmed in primary adult CMs isolated from ucp3^−/−^ or WT mice (Figure 2A through 2C). Taken together, these results suggest that ucp3 levels regulate mitochondrial function, ROS production, and, in turn, cell survival under hypoxic conditions in vitro.

### Mitochondria Respiratory Parameters of WT and ucp3^−/−^Mice Are Differently Affected by Ischemia–Reperfusion

**Figure 3.**
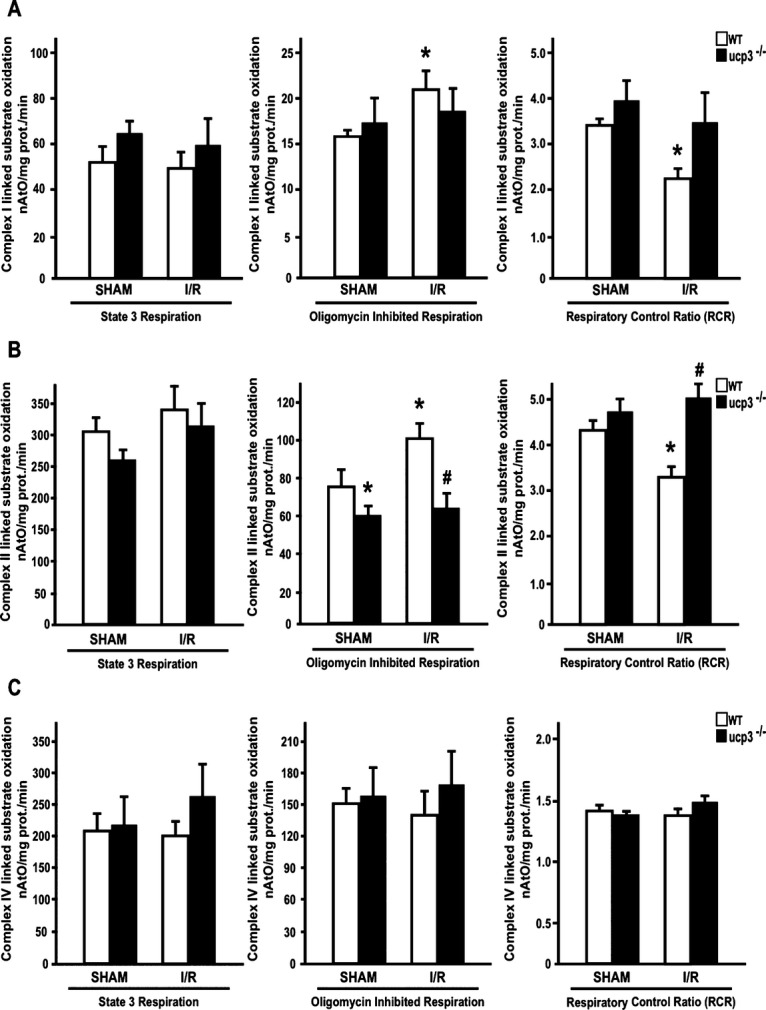
The ucp3 genetic deletion affects mitochondria respiratory parameters in ischemic–reperfused hearts. A, Complex I linked substrate for mitochondrial state 3 (excess ADP, pyruvate, and malate), state 4 (oligomycin‐treated) respiration (nAtO_2_/mg prot./min) and respiratory control ratio (RCR=state 3/state 4) of ucp3^−/−^ and WT hearts (n=6 mice for group, *P<0.05 for unpaired Student *t* test comparing SHAM and I/R of different genotype; *P<0.05 vs SHAM WT). B, Complex II linked substrate for mitochondrial state 3 (excess ADP, pyruvate, and malate), state 4 (oligomycin‐treated) respiration (nAtO_2_/mg prot./min) and respiratory control ratio (RCR=state 3/state 4) of ucp3^−/−^ and WT hearts (n=6 mice for group, *P<0.05 and #*P*<0.05 for unpaired Student *t* test comparing SHAM and I/R of different genotype; *P<0.05 vs SHAM WT, #*P*<0.05 vs I/R WT). C, Complex IV linked substrate for mitochondrial state 3 (excess ADP, pyruvate, and malate), state 4 (oligomycin‐treated) respiration (nAtO_2_/mg prot./min) and respiratory control ratio (RCR=state 3/state 4) of ucp3^−/−^ and WT hearts (n=6 mice for group, not significant). ucp3 indicates uncoupling protein 3; I/R, ischemia–reperfusion; WT, wild‐type; SHAM, sham‐operated control animals.

To detect how ucp3 deletion and ischemia–reperfusion might affect mitochondrial respiratory parameters, oxygen consumption was evaluated in experimental conditions in which the synthesis and the export of ATP are at maximal rates (state 3_ADP_) and when this process is terminated by oligomycin (state 4_O_). In addition, we measured the respiratory control ratio (RCR). Under normoxic conditions, the absence of ucp3 significantly influenced the mitochondrial respiration rate only when a complex II–linked substrate was used (Figure 2A through 2C). Indeed, succinate (+rotenone)‐energized mitochondria from ucp3^−/−^ mice showed a significant reduction in state 4_O_ respiration, whereas no significant differences in state 3_ADP_ respiration values were observed (Figure 3A through 3C). At the same time, the RCR value was higher in ucp3^−/−^ mice compared with WT mice (Figure 3A through 3C).

In mitochondria from WT mice, ischemia–reperfusion induced an increase in state 4 respiration and a decrease in RCR values, index of mitochondrial uncoupling, when using glutamate+malate and succinate+rotenone as substrates. Interestingly, while ischemia and reperfusion affected mitochondrial parameters in WT mice, it did not affect them in mitochondria from knockout mice (Figure 3A through 3C). Finally, the absence of neither ucp3 nor ischemia–reperfusion affected mitochondria respiratory parameters detected using a complex IV–linked substrate, since no significant differences in state 3_ADP_, state 4_O_, and RCR were observed in any considered group. Taken together, these data confirm that ischemia–reperfusion induces mitochondrial uncoupling, as already reported in the literature,^35^ and suggest that ucp3 might be involved in ischemia–reperfusion–induced uncoupling.

### Myocardial Infarct Size Is Increased in ucp3^−/−^ Mice After Permanent Coronary Ligation

**Figure 4.**
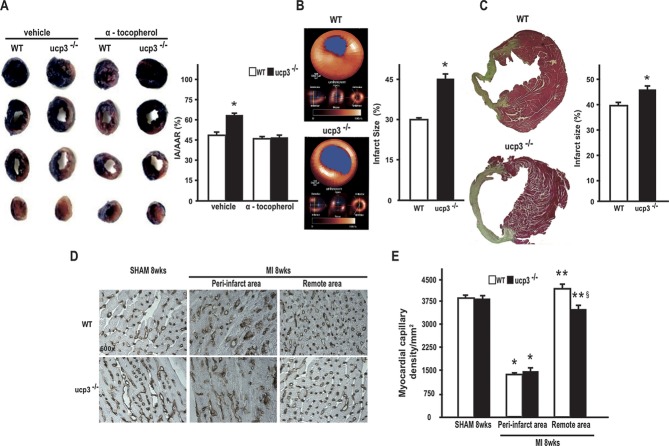
The ucp3 genetic deletion increases infarct size after MI. A, Left: Representative images of TTC staining of heart sections from WT+vehicle, ucp3^−/−^+vehicle, WT+α‐tocopherol, and ucp3^−/−^+α‐tocopherol mice after MI. Right: Bar graphs showing ratios of myocardial infarct area vs area at risk (IA/AAR) in both groups (*P<0.05 for 2‐way ANOVA test comparing vehicle and α‐tocopherol of different genotype; *P<0.05 vs all, n=7 for MI+vehicle and n=4 for MI+α‐tocopherol). B, Left: Representative positron emission tomography with 2‐deoxy‐2[^18^F]fluoro‐d‐glucose (^18^F‐FDG PET) horizontal long‐axis slice from anterior to inferior wall, long‐axis slice from apex to base, and short‐axis slice from anterior to inferior wall from WT and ucp3^−/−^ mice after MI. Right: Quantitative analysis of infarct size defined as the proportion of myocardial contour area of infarcted myocardium, derived by PET (*P<0.05 for unpaired Student *t* test comparing MI of different genotype; *P<0.05 vs WT n=14 hearts/group). C, Left: Representative images of Masson's trichrome staining of heart sections from WT and ucp3^−/−^ mice after MI. Right: Bar graphs showing percent infarct size (*P<0.05 for unpaired Student *t* test comparing MI of different genotype; *P<0.05 vs WT n=10 hearts/group). D, Representative lectin staining in cardiac sections from WT SHAM (n=4), ucp3^−/−^ SHAM (n=4), WT MI (n=6), ucp3^−/−^ MI (n=6) mice. Peri‐infarct area and remote area are shown. Capillaries appear brown (×600 magnification). E, Cumulative data of multiple independent experiments analyzing capillarity density (*P<0.05 for 2‐way ANOVA test comparing MI peri‐infarct area to SHAM; **P<0.05 for 2‐way ANOVA test comparing MI remote area to MI peri‐infarct area; ^§^*P*<0.05 for 2‐way ANOVA comparing ucp3^−/−^ MI remote area to WT MI remote area; *P<0.05 vs SHAM; **P<0.05 vs MI peri‐infarct area; §*P*<0.05 vs WT MI remote area). ucp3 indicates uncoupling protein 3; WT, wild‐type; MI, myocardial infarction; TTC, triphenyltetrazolium chloride; ANOVA, analysis of variance; PET, positron emission tomography; SHAM, sham‐operated control animals.

To test whether ucp3 genetic deletion might affect cardiac responses to ischemia in vivo, MI was induced by permanent coronary artery ligation in WT and ucp3^−/−^ mice as previously described.^28^ Sham‐operated animals from both genotypes underwent the same surgical procedure without occlusion of the coronary artery. Because ucp3 levels regulate cell survival under hypoxic conditions in vitro, studies were performed to determine whether ucp3 levels might affect the mean infarct size after permanent coronary artery ligation. Twenty‐four hours after permanent coronary artery ligation, hearts were infused with Evans blue to demarcate the ischemic area susceptible to infarction (AAR), and counterstained with triphenyltetrazolium chloride to identify the final IA from the viable myocardium within the AAR. Despite similar AARs between the 2 groups (Table 1), ucp3^−/−^ hearts demonstrated a significantly larger proportion of infarct myocardium within the AAR compared with WT mice (Figure 4A and Table 1). Interestingly, treatment with the antioxidant α‐tocopherol significantly reduced the infarct size in ucp3^−/−^ mice but did not exert any effects in WT MI mice (Figure 3A and Table 1), suggesting that increased ROS production might be responsible for the larger IAs in ucp3^−/−^ hearts.

**Table 1.** IA and AAR After 24 Hours of MI in WT and ucp3^−/−^ Mice

	MI+Vehicle		MI+α‐Tocopherol	
	WT (n=7)	ucp3^−/−^ (n=7)	WT (n=5)	ucp3^−/−^ (n=4)
IA/AAR, %	48.2±3.7	65.0±2.9*	46.4±3.7	47.0±2.0
AAR/LV, %	35.1±1.2	29.6±3.3	28.9±3.9	31.3±5.4

MI indicates myocardial infarction; WT, wild‐type; ucp3, uncoupling protein 3; IA, infarct area; AAR, area at risk; LV, left ventricle area.

* *P*<0.05 vs WT MI+vehicle.

Eight weeks after MI, ucp3^−/−^ hearts still displayed a significantly larger infarct size by positron emission tomography imaging (Figure 4B) and Masson's trichrome staining (Figure 4C). To rule out abnormal vascular density as the cause of increased infarct size in ucp3^−/−^ mice, histological studies were performed to determine capillary density in WT and ucp3^−/−^ hearts before and after MI. WT and ucp3^−/−^ mice displayed similar capillary densities under basal conditions and, after 8 weeks MI, in the peri‐IA (Figure 4D and 4E). Interestingly, ucp3^−/−^ mice showed a significant decrease of the capillary density in the remote area after 8 weeks of MI compared with WT mice (Figure 4D and 4E).

### Genetic Deletion of the ucp3 Gene Reduces Survival and Cardiac Function After Acute MI

**Figure 5.**
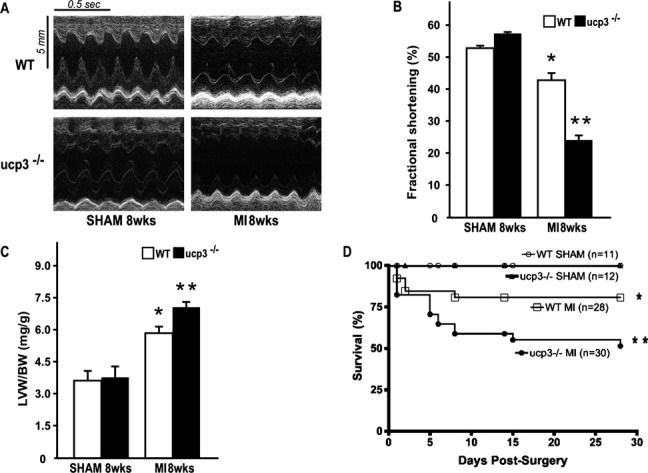
ucp3 genetic deletion promotes cardiac dysfunction and reduces survival after MI. A, Representative M‐mode echocardiographic tracings from WT and ucp3^−/−^ mice after 8 weeks MI or SHAM procedure. B, Cumulative data of % fractional shortening (FS) of WT and ucp3^−/−^ mice 8 weeks after MI (*P<0.05 and **P<0.05 for 2‐way ANOVA test comparing SHAM and MI of different genotype; *P<0.05 vs SHAM; **P<0.05 vs SHAM, WT MI n=10 hearts/group). C, Bar graphs showing left ventricle weight/body weight (LVW/BW) ratios in WT and ucp3^−/−^ mice after MI or SHAM procedure (*P<0.05 and **P<0.05 for 2‐way ANOVA test comparing SHAM and MI of different genotype; *P<0.05 vs SHAM; **P<0.05 vs SHAM, WT MI n=10 hearts/group). D, Kaplan–Meier cumulative survival analysis of WT and ucp3^−/−^ mice after MI (WT: SHAM, n=11, MI, n=28; ucp3^−/−^: SHAM, n=12, MI, n=30; *P<0.001 and **P<0.001 comparing MI to SHAM of different genotype, *P<0.001 vs SHAM, ** *P*<0.001 vs SHAM, WT MI). ucp3 indicates uncoupling protein 3; WT, wild‐type; MI, myocardial infarction; ANOVA, analysis of variance; SHAM, sham‐operated control animals.

Infarct size is one of the major determinants of postischemic cardiac remodeling. To evaluate cardiac function in WT and ucp3^−/−^ mice after MI, transthoracic echocardiography was performed in all experimental groups before and 8 weeks after MI. Under basal conditions, ucp3^−/−^ mice displayed normal cardiac dimensions and function (Figure 5A and 5B and Table 2). As expected, both WT and ucp3^−/−^ mice developed cardiac dysfunction after MI (Figure 5A and 5B and Table 2). However, compared with WT, ucp3^−/−^ mice displayed a significant worsening in cardiac function after coronary artery ligation and a more pronounced increase in the LV weight–to–body weight ratio at study termination (Figure 5C and Table 2).

**Table 2.** Morphometric and Echocardiographic Parameters After 8 Weeks of MI or Sham Operation in WT and ucp3^−/−^ Mice

	SHAM 8 Weeks		MI 8 Weeks	
	WT (n=11)	ucp3^−/−^ (n=12)	WT (n=30)	ucp3^−/−^ (n=28)
Morphometry				
BW, g	26±0.9	25.8±0.8	26±0.2	25.3±0.4
LVW, mg	98±5.4	100.1±1.1	138.2±13.5*	160.8±12.8*
LVW/BW, mg/g	3.7±0.6	3.8±0.47	5.5±1.0*	6.8±1.0*
Echocardiography				
LVEDD, mm	3.4±0.03	3.4±0.06	4.1±0.8*	5.1±0.04*
LVESD, mm	1.6±0.02	1.4±0.03	2.4±0.2*	3.8±0.05*
FS, %	54.0±0.02	58.8±0.02	42.7±0.03*	24.4±0.05*
IVSd, mm	0.9±0.08	0.8±0.12	1.1±0.10	1.01±0.05
PWd, mm	0.9±0.08	0.93±0.12	0.8±0.10	0.9±0.05
HR, bpm	457±24	469±15	486±20	479±22

MI indicates myocardial infarction; SHAM, sham‐operated control animals; WT, wild‐type; ucp3, uncoupling protein 3; BW, body weight; LVW, left ventricular weight; LVEDD, left ventricular end‐diastolic diameter; LVESD, left ventricular end‐systolic diameter; FS, fractional shortening; IVSd, end‐diastolic interventricular septum; PWd, end‐diastolic posterior wall; HR, heart rate.

* *P*<0.05 vs SHAM.

* *P*<0.05 vs SHAM and WT MI.

To assess whether these differences would translate into a survival benefit, survival rates were monitored for 30 days in all the groups after the surgery. As expected, survival of the sham 8‐week groups was 100% (Figure 5D), whereas MI resulted in a significantly lower survival in both genotypes compared with the respective sham 8‐week group (*P*<0.05). However, the 30‐day survival of ucp3^−/−^ MI mice was significantly lower compared with WT MI mice (56 versus 85, respectively; *P*<0.05; Figure 5D).

### ucp3 Genetic Deletion Promotes Mitochondrial Dysfunction and Exaggerates Mitochondrial ROS Production After Coronary Artery Ligation

**Figure 6.**
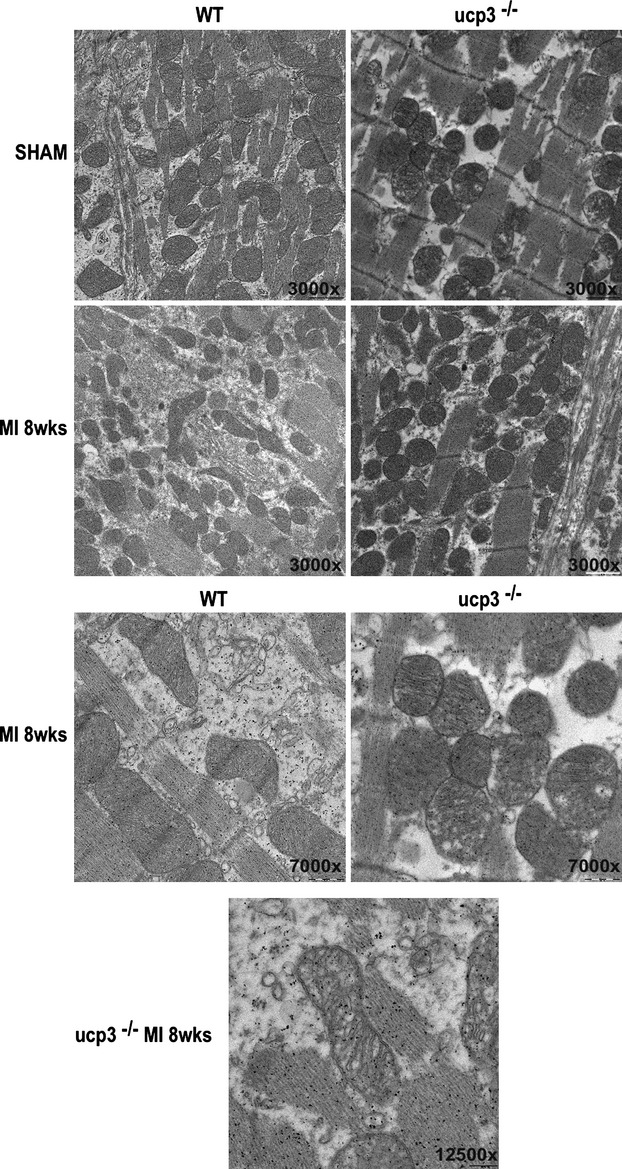
ucp3 genetic deletion promotes cardiac mitochondria morphological changes. Representative electron microscopy microphotographs of cardiac sections from WT and ucp3^−/−^hearts after SHAM or MI procedure (×3000, ×7000, and ×12 500 magnification; n=6 hearts/group). ucp3 indicates uncoupling protein 3; WT, wild‐type; MI, myocardial infarction; SHAM, sham‐operated control animals.

**Figure 7.**
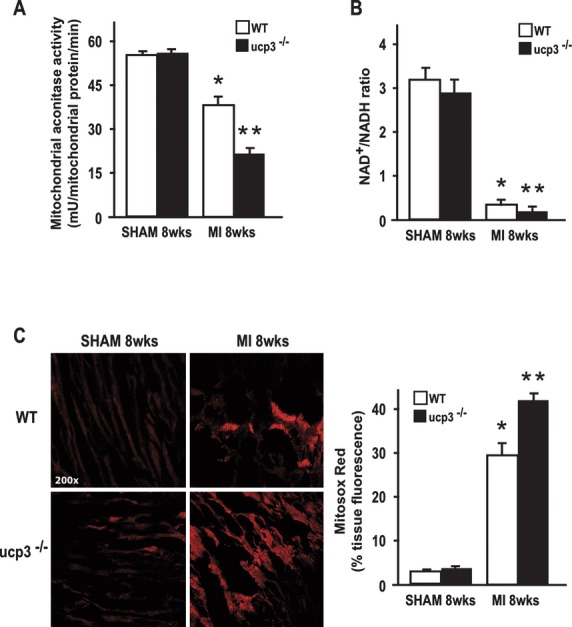
ucp3 genetic deletion promotes mitochondrial dysfunction in vivo. A, Mitochondrial aconitase activity in WT and ucp3^−/−^ hearts after MI (*P<0.05 and **P<0.05 for 2‐way ANOVA test comparing SHAM and MI of different genotype; *P<0.05 vs SHAM; **P<0.05 vs SHAM, WT MI n=5 hearts/group). B, NAD^+^/NADH ratio in WT and ucp3^−/−^ hearts after SHAM or MI procedure (*P<0.05 and **P<0.05 for 2‐way ANOVA test comparing SHAM and MI of different genotype; *P<0.05 vs SHAM; **P<0.05 vs SHAM, WT MI n=5 hearts/group). C, Left: Representative Mitosox staining of cardiac sections of WT and ucp3^−/−^ hearts after MI (×200 magnification). Right: Cumulative data of Mitosox fluorescence from independent experiments (*P<0.05 and **P<0.05 for 2‐way ANOVA test comparing SHAM and MI of different genotype; *P<0.05 vs SHAM; **P<0.05 vs SHAM, WT MI n=5 hearts/group). ucp3 indicates uncoupling protein 3; WT, wild‐type; MI, myocardial infarction; ANOVA, analysis of variance; SHAM, sham‐operated control animals.

To analyze the effects of ucp3 genetic deletion on mitochondrial structure and function, electron microscopy studies were performed on WT and ucp3^−/−^ MI hearts. Interestingly, under basal conditions, mitochondria of both WT and ucp3^−/−^ CMs were morphologically normal (Figure 6). After MI, mitochondria of WT CMs were dilated with well‐defined lamellar cristae and clear matrix (Figure 5). Conversely, after coronary ligation in ucp3^−/−^ mice, a dense mitochondrial matrix could be observed within the dilated cristae (Figure 6). Moreover, in ucp3^−/−^ MI hearts, diverticula‐like protrusions could be seen from the mitochondrial wall, suggesting the occurrence of organelle fragmentation that ultimately could justify the apparent increase in number of small mitochondria (Figure 6). These morphological abnormalities were associated with reduced mitochondrial aconitase activity and NAD^+^/NADH ratio in ucp3^−/−^ MI hearts compared with WT MI hearts (Figure 7A and 7B), whereas mitochondrial ROS generation was significantly increased (Figure 7C).

### Increased Apoptotic Cell Death in ucp3^−/−^ Hearts After Coronary Artery Ligation

**Figure 8.**
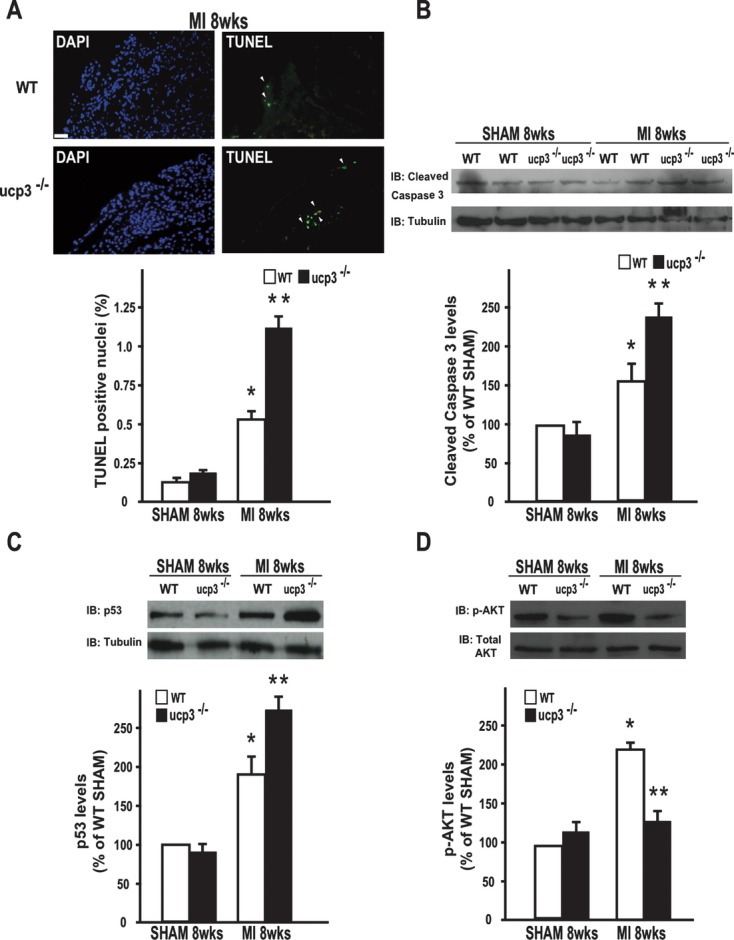
ucp3 genetic deletion and cardiomyocyte death in vivo. A, Top: Representative DAPI (left) and TUNEL staining (right) in cardiac sections of WT and ucp3^−/−^ hearts after 8 weeks MI. Positive nuclei appear green (arrowheads). Bottom: Cumulative data of multiple independent experiments (*P<0.05 and **P<0.05 for 2‐way ANOVA test comparing SHAM and MI of different genotypes; *P<0.05 vs SHAM; **P<0.05 vs SHAM, WT MI n=5 hearts/group). Scale bar=250 μm. Representative immunoblot (IB) (top) and densitometric analysis (bottom) of 4 independent experiments to evaluate cleaved caspase 3 (B), p53 (C), and phosphorylated‐AKT (D) protein levels in WT and ucp3^−/−^ hearts after MI (for all, *P<0.05 and **P<0.05 for 2‐way ANOVA test comparing SHAM and MI of different genotypes; *P<0.05 vs SHAM; **P<0.05 vs SHAM, WT MI n=5 hearts/group). ucp3 indicates uncoupling protein 3; WT, wild‐type; MI, myocardial infarction; DAPI, 4′‐6‐diamidino‐2‐phenylindole; ANOVA, analysis of variance; SHAM, sham‐operated control animals.

To determine whether mitochondrial dysfunction in ucp3^−/−^ mice would increase apoptotic cell death after MI, TUNEL staining was performed in cardiac sections from the different groups. As shown in Figure 8A, ucp3 genetic deletion significantly increased the rate of apoptotic cell death 8 weeks after MI. Consistent with these results, cleaved caspase 3 and p53 levels were significantly higher in ucp3^−/−^ hearts after MI, whereas AKT phosphorylation was significantly reduced (Figure 8B through 8D). Taken together, these data suggest that ucp3 levels regulate cell survival and infarct size after acute MI by modulating mitochondrial structural and functional adaptations to myocardial ischemia.

## Discussion

The present study shows that mitochondrial ucp3 plays a pivotal role in the regulation of mitochondrial function, ROS production, and cell survival in the ischemic heart, modulating infarct size, cardiac remodeling, and survival after MI. These beneficial effects could be related to the ability of ucp3 to promote mitochondrial uncoupling and control ROS production and suggest that uncoupling proteins might represent novel therapeutic targets in patients with coronary artery disease.

In patients with an acute ischemic event, the prognosis is mainly dependent on the amount of myocardium that is lost.^36^ Thus, during the past 40 years, there has been an intense effort to identify cardioprotective therapies to limit infarct size and ameliorate cardiac remodeling.^37–38^ There is no definitive evidence that antioxidants exert a beneficial effect on postischemic cardiac remodeling in clinical trials,^9^ and it is possible that clinically available drugs are insufficient in their potency, the dose of the drug is not optimal, or the timing of administration is not appropriate.

The family of mitochondrial uncoupling proteins has been recently recognized as being important in the regulation of mitochondrial function and ROS production.^39^ In the mammalian heart, ucp2 and ucp3 are the predominant ucp isoforms,^16^ and there is substantial literature supporting the protective effect of these ucps in ischemia–reperfusion injury.^40–41^ Indeed, overexpression of UCPs decreases ROS production, improves CM survival and contractile function in the setting of ischemia–reperfusion. However, the effects of ucp3 deletion under conditions of chronic HF induced by permanent coronary artery ligation have never been tested. Importantly, while a decrease in ucp3 expression would be expected to enhance the energetic efficiency in stressed CMs, it might also have the counterbalancing effect of allowing greater production of ROS. Thus, the question remains of whether ucp3 downregulation in chronic HF is maladaptive.

Our study shows that ucp3 deletion tends to increase oxidative phosphorylation efficiency, since higher RCR values were observed in ucp3^−/−^ mice compared with WT mice. In addition, results indicate that ucp3 is possibly involved in ischemia–reperfusion–induced uncoupling. In fact, the increase of mitochondrial respiration principally controlled by the activity of the proton leak (state 4_O_) and the decrease in RCR are observed solely in mitochondria from WT mice.

After ischemia–reperfusion, the activation of UCP3‐mediated‐uncoupling could be considered as a mechanism of protection against ROS damage. This concept is in line with data indicating that the administration of low concentrations of uncoupling agents, such as carbonyl cyanide *p*‐(trifluoromethoxy)phenylhydrazone (FCCP) or 2,4‐dinitrophenol, evokes protection against ischemic damage in the intact heart,^42^ in CMs,^43^ and in the brain,^44^ in parallel with a diminution in mitochondrial ROS production. Consistent with this assumption, our study shows that ucp3 expression levels modulate cell survival under low oxygen conditions in vitro and in vivo, because ucp3 genetic deletion exaggerated hypoxia or ischemia‐induced apoptotic cell death.

Interestingly, ucp3^−/−^ hearts displayed increased levels of oxidative damage markers and decreased activity of aconitase, a protein sensitive to damage by superoxide.^45^ In ucp3^−/−^ hearts, such abnormalities were associated with larger IAs after permanent coronary artery occlusion, adverse remodeling, and reduced survival. Importantly, in vivo treatment with the antioxidant α‐tocopherol reduced the infarct size in ucp3^−/−^ mice to WT values, suggesting that ucp3 deletion may enhance cell death in response to hypoxia by increasing ROS production over WT levels. However, unlike 2,4‐dinitrophenol,^42^ α‐tocopherol did not reduce the infarct size in WT mice, confirming results from clinical trials showing that inhibition of endogenous ROS levels is not sufficient to prevent cell death induced by myocardial ischemia^9^ and suggesting that other mechanisms triggered by mitochondrial uncoupling might regulate CM survival under low oxygen conditions. Indeed, in addition to the regulation of ROS production, it has been suggested that ucp2 and/or ucp3 proteins are essential components of the mitochondrial calcium uptake pathway,^46^ because overexpression of these proteins enhanced the transfer of cytosolic calcium signals to the mitochondria, which could also protect the cells from oxidative stress.^46^ However, this possible novel function of ucp2 and ucp3 has been subsequently challenged.^47^ Indeed, recent results indicate that ucp3 is not a mitochondrial Ca^2+^ uniporter and that it negatively modulates the activity of sarcoplasmic/endoplasmic reticulum Ca^2+^ pumps by limiting mitochondrial ATP production.^48^ Thus, the effects of ucp3 on mitochondrial Ca^2+^ seem to merely reflect metabolic alterations that have an impact on cellular Ca^2+^ homeostasis.

**Figure 9.**
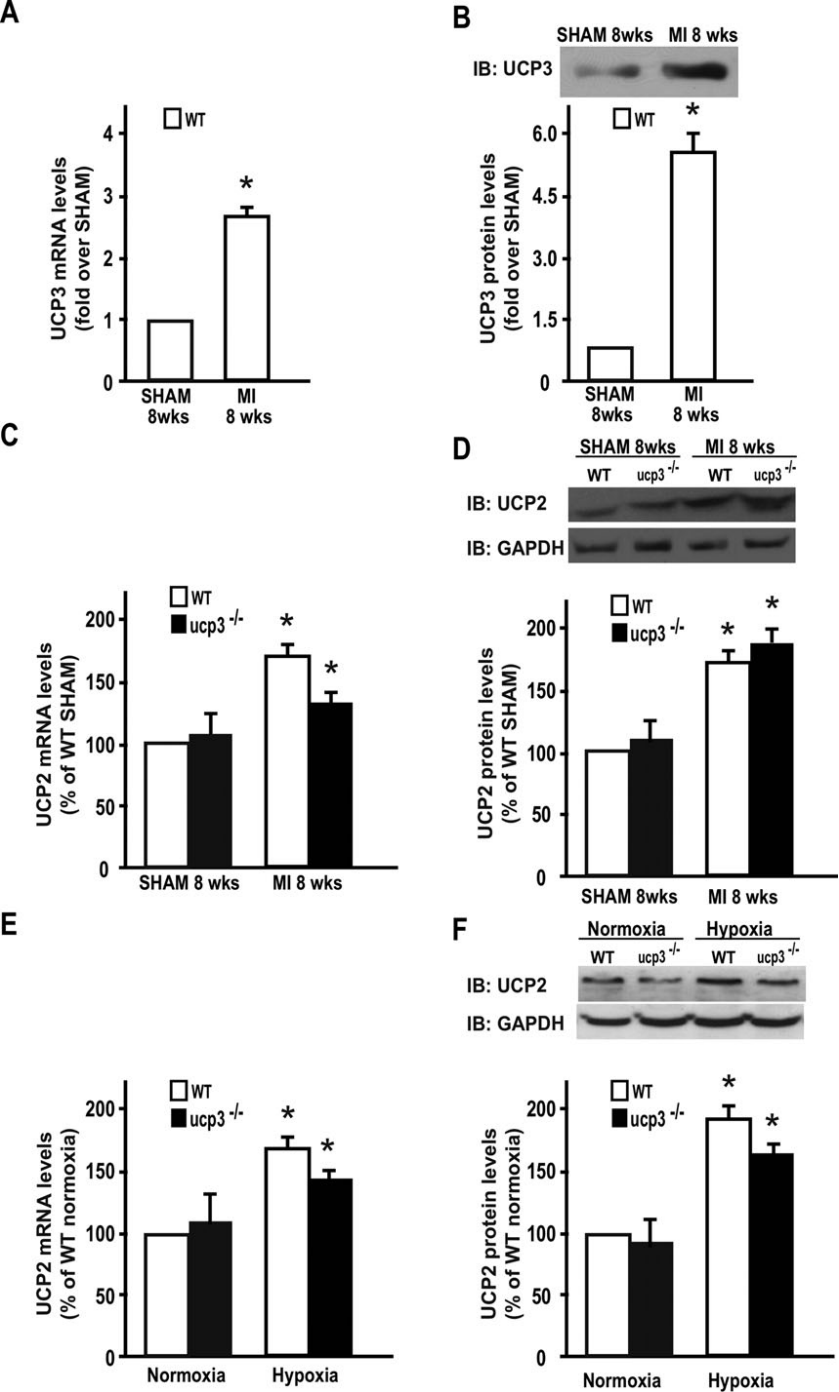
UCP2 and UCP3 levels in hearts and MEFs from WT and ucp3^−/−^ mice. A, ucp3 mRNA levels in WT and ucp3^−/−^ hearts after 8 weeks of myocardial infarction (*P<0.05 for unpaired Student *t* test comparing MI to SHAM; n=5 hearts/group). B, Representative immunoblot (IB) and densitometric analysis of 4 independent experiments to evaluate ucp3 protein levels in WT hearts after 8 weeks of myocardial infarction (*P<0.05 for unpaired Student *t* test comparing MI to SHAM; n=5 hearts/group). C, ucp2 mRNA levels in WT and ucp3^−/−^ hearts after 8 weeks of myocardial infarction (*P<0.05 for 2‐way ANOVA test comparing SHAM and MI of different genotype; *P<0.05 vs SHAM n=5 hearts/group). D, Representative IB and densitometric analysis of 4 independent experiments to evaluate ucp2 protein levels in WT and ucp3^−/−^ hearts after 8 weeks of myocardial infarction (*P<0.05 for 2‐way ANOVA test comparing SHAM and MI of different genotype; *P<0.05 vs SHAM n=5 hearts/group). E, ucp2 mRNA levels in WT and ucp3^−/−^ MEFs during normoxic and hypoxic conditions (*P<0.05 for 2‐way ANOVA test comparing normoxia and hypoxia of different genotype; *P<0.05 vs normoxia). F, Representative IB and densitometric analysis of 4 independent experiments to evaluate ucp2 protein levels in WT and ucp3^−/−^ MEFs during normoxic and hypoxic conditions (*P<0.05 for 2‐way ANOVA test comparing normoxia and hypoxia of different genotypes; *P<0.05 vs normoxia). ucp indicates uncoupling protein; MEFs, murine embryonic fibroblasts; WT, wild‐type; MI, myocardial infarction; ANOVA, analysis of variance; SHAM, sham‐operated control animals.

After 8 weeks of MI, ucp3^−/−^ hearts were also characterized by reduced capillary density in the remote peri‐IA of the left ventricle. These data suggest that ucp3 deletion might exert detrimental effects on cardiac function and remodeling of the ischemic heart via other mechanisms, eventually acting in surviving cells, including vascular rarefaction.^25,49^ Importantly, MI hearts from ucp3^−/−^ mice were characterized by structural and functional mitochondrial abnormalities and increased mitochondrial ROS production despite a normal induction of ucp2 mRNA/protein levels in vitro and in vivo (Figure 9A through 9F).

In addition to hemodynamic alterations and HF,^50^ doxorubicin treatment has been shown to decrease ucps expression.^51–52^ Thus, although our study focused on the role of ucp3 during conditions of myocardial ischemia, it is also possible that this protein might be involved in the pathogenesis of different pathological cardiac conditions, such as those induced by cardiotoxic chemotherapy drugs.

In conclusion, our study indicates a role for ucp3 in ischemic failing murine heart. The ucp3 genetic deletion promotes mitochondrial dysfunction, and increases ROS production and apoptotic cell death under low oxygen conditions, enlarging infarct size and accelerating HF. These results suggest that ucp3 levels might represent a novel determinant of postischemic cardiac remodeling and survival.
